# Perception of Quality of Life, Brain Regions, and Cognitive Performance in Hispanic Adults: A Canonical Correlation Approach

**DOI:** 10.3390/ctn9030033

**Published:** 2025-07-23

**Authors:** Juan C. Lopez-Alvarenga, Jesus D. Melgarejo, Jesus Rivera-Sanchez, Lorena Velazquez-Alvarez, Isabel Omaña-Guzmán, Carlos Curtis-Lopez, Rosa V. Pirela, Luis J. Mena, John Blangero, Jose E. Cavazos, Michael C. Mahaney, Joseph D. Terwilliger, Joseph H. Lee, Gladys E. Maestre

**Affiliations:** 1 Division of Population Health & Biostatistics, School of Medicine, University of Texas Rio Grande Valley, Edinburg, TX 78539, USA; 2 Rio Grande Valley Alzheimer’s Disease Resource Center for Minority Aging Research, University of Texas Rio Grande Valley, Brownsville, TX 78520, USA; 3 School of Medicine, Universidad Mexico Americana del Norte, Reynosa 88640, Mexico; 4 Institute of Neuroscience, School of Medicine, University of Texas Rio Grande Valley, Harlingen, TX 78550, USA; 5 Departament of Geriatrics, Hospital General de México “Dr. Eduardo Liceaga”, Mexico City 06720, Mexico; 6 Hospital Angeles Roma, Mexico City 06760, Mexico; 7 Pediatric Obesity Clinic and Wellness Unit, Hospital General de México “Dr. Eduardo Liceaga”, Mexico City 06720, Mexico; 8 Trauma & Orthopaedics, Royal London Hospital, Barts Health NHS Trust, London E1 1BB, UK; 9 The South Texas Alzheimer’s Disease Research Center, University of Texas Health Science Center at San Antonio, San Antonio, TX 78229, USA; 10 Department of Informatics, Universidad Politécnica de Sinaloa, Mazatlán 82124, Mexico; 11 Department of Human Genetics, University of Texas Rio Grande Valley, Brownsville, TX 78520, USA; 12 South Texas Diabetes and Obesity Institute University of Texas Rio Grande Valley, Brownsville, TX 78520, USA; 13 Department of Neurology, Glenn Biggs Institute for Alzheimer’s & Neurodegenerative Diseases, University of Texas Health Science Center at San Antonio, San Antonio, TX 78229, USA; 14 Departments of Psychiatry and Genetics & Development, Columbia University Medical Center, New York, NY 10032, USA; 15 Gertrude H. Sergievsky Center, Columbia University Medical Center, New York, NY 10032, USA; 16 Division of Medical Genetics, New York State Psychiatric Institute, New York, NY 10032, USA; 17 Division of Public Health Genomics, National Institute for Health and Welfare, 00271 Helsinki, Finland; 18 Taub Institute for Research on Alzheimer’s Disease and the Aging Brain, Columbia University, New York, NY 10032, USA; 19 Department of Neurology, College of Physicians and Surgeons, Columbia University, New York, NY 10032, USA; 20 Department of Epidemiology, Joseph P. Mailman School of Public Health, Columbia University, New York, NY 10032, USA

**Keywords:** brain parcellation, magnetic resonance imaging, quality of life, Hispanics

## Abstract

The quality of life (QoL) perception has been studied in neurological diseases; however, there is limited information linking brain morphological characteristics, QoL, and cognition. Human behavior and perception are associated with specific brain areas that interact through diffuse electrochemical networking. We used magnetic resonance imaging (MRI) to analyze the brain region volume (BRV) correlation with the scores of Rand’s 36-item Short Form Survey (SF-36) and cognitive domains (memory and dementia status). We analyzed data from 420 adult participants in the Maracaibo Aging Study (MAS). Principal component analysis with oblimin axis rotation was used to gather redundant information from brain parcels and SF-36 domains. Canonical correlation was used to analyze the relationships between SF-36 domains and BRV (adjusted for intracranial cavity), as well as sex, age, education, obesity, and hypertension. The average age (±SD) of subjects was 56 ± 11.5 years; 71% were female; 39% were obese; 12% had diabetes, 52% hypertension, and 7% dementia. No sex-related differences were found in memory and orientation scores, but women had lower QoL scores. The 1st and 2nd canonical correlation roots support the association of SF-36 domains (except social functioning and role emotional) and total brain volume, frontal lobe volume, frontal pole, lateral orbital lobe, cerebellar, and entorhinal areas. Other variables, including age, dementia, memory score, and systolic blood pressure, had a significant influence. The results of this study demonstrate significant correlations between BRV and SF-36 components, adjusted for covariates. The frontal lobe and insula were associated with the mental health component; the lateral-orbital frontal lobe and entorhinal area were correlated with the physical component.

## 1. Introduction

Quality of life (QoL) is a multidimensional construct that connects individuals’ perceptions of their physical health, psychological state, level of independence, social relationships, and their relationship to salient features of the environment. In clinical and public health research, QoL assessment tools are commonly used to compute quality-adjusted life years (QALYs), which help estimate cost-utility for interventions including treatments, diagnostic tools, or health system processes. For example, utility and quality-adjusted survival have been used to evaluate the 5-year impact of stroke and TIA [1], or the public impact of thrombectomy was evaluated with QALYs to provide information on its efficiency [2].

QoL instruments typically measure self-perceived well-being, which is, in turn, associated with brain function and structure. Interactions between specific functional and anatomical brain areas modulate behavior and cognition [3–5]. For instance, the hippocampus plays a role in forming new memories of events and facts, and the amygdala is involved in context-dependent memory consolidation [6]. Other brain areas, including the cerebellum, coordinate fine motor control and procedural memory learning. Although different types of memory (short-term, sensory, and long-term) are thought to be stored in neural networks in various parts of the brain, the physical location of memories remains partially understood [7,8].

QoL is not only a measure of physical health but also includes self-perception and cognitive interpretation, which are memory-related brain functions. The hippocampus and entorhinal cortex play roles in episodic memory, contextualizing previous events, and predicting future events. These functions are creating goals and achieving happiness in life [9]. A recent study showed that QoL is associated with the default mode network, suggesting the complex elaboration of self-wellness perception [10].

High-resolution magnetic resonance imaging (MRI) offers detailed visualization of anatomical areas and has been utilized to detect psychiatric disorders and cognitive decline [11]. Functional MRI in individuals with mild cognitive impairment has revealed reduced fractional anisotropy values in the posterior cingulate gyrus, parahippocampal gyrus, thalamus, and caudate in the left hemisphere, as well as in the bilateral precuneus [12].

Studying the association between brain structure and perceived well-being through epidemiological research is complex, partly due to cultural variation in behavior and perception. Recent studies have correlated brain structure with intelligence [13], subjective well-being, and dispositional hope (the tendency to plan and meet goals), as well as psychological traits that contribute to individuals’ life satisfaction and evaluation [14].

In this study, we investigated the relationship between broad brain regions and quality of life (QoL), which indicates health in elderly individuals [15]. Interestingly, the perception of QoL is intrinsically linked to sex and brain functioning. In older adults, cultural values and social support structures play a critical role in how individuals evaluate their well-being. For instance, English-speaking Hispanics over the age of 50 appear to be more satisfied with their lives than non-Hispanic whites, despite living in more adverse circumstances and having higher rates of chronic illnesses. This phenomenon is called “the Hispanic paradox” and is thought to be partially attributed to strong family and community cohesion, spiritual beliefs, and cultural resilience [16].

To the best of our knowledge, this is among the first studies to use canonical correlation analysis to simultaneously assess the multivariate relationships among brain region volumes, cognitive tests, clinical variables, and QoL dimensions in aging Hispanic adults. Specifically, we hypothesized that the frontal cortex (involved in emotional regulation and complex learning) [17], the hippocampus (involved in memory and reading) [18], and the cerebellum (involved in cognitive and affective behavior processes) [19,20] would show significant associations with the QoL dimensions. This study contributes to a growing body of research aimed at understanding the interaction between brain structure and subjective well-being in this underrepresented population.

## 2. Materials and Methods

### 2.1. Study Sample

This is a cross-sectional secondary analysis of the Maracaibo Aging Study (MAS), a longitudinal population-based study conducted in Santa Lucía and Santa Rosa de Agua, two communities approximately 7 km apart, sharing similar cultural, social, and educational backgrounds. The MAS was focused on assessing cognitive disorders with a methodology that has been described elsewhere [21]. The MAS had enrolled 2439 subjects, but not all had information on MRI. The current analysis included 420 participants aged 40 years or older who had data on brain MRI, cognitive testing, and QoL assessments. All participants signed an informed consent form approved by the Institutional Review Boards of the Cardiovascular Institute at the University of Zulia, Maracaibo, and Columbia University, New York. The current study is a secondary analysis of the MAS, involving no new data collection. The IRB of the University of Zulia approved the study in 1997 in collaboration with the IRB of Columbia University. The Institutional Review Board of the University of Texas Rio Grande Valley approved the secondary analysis in 2025. The original and this secondary analysis followed the principles of the Declaration of Helsinki.

### 2.2. Demographic and Clinical Characteristics

Physicians and trained nurses collected demographic information (age, sex, education) and clinical data (anthropometry, blood pressure, clinical history). Obesity was defined as a body mass index (BMI) of greater than 30 kg/m^2^. Type 2 diabetes mellitus (T2DM) was diagnosed based on current ADA criteria [22]. Blood pressure was assessed using 24 h ambulatory blood pressure monitoring (SpaceLabs Inc., Redmond, WA, USA), programmed to record measurements every 15 min during waking hours (6:00–23:00 h) and every 30 min during sleep (23:00–6:00 h).

### 2.3. Cognitive Assessment

The neuropsychiatric assessment included Mini-Mental State Examination (MMSE) [23,24], the Blessed Orientation-Memory and Concentration Test [25], and the Neuropsychiatric Inventory [26]. Dementia diagnoses were based on data collected by trained social workers through in-home interviews using the Dementia Questionnaire [26], the Blessed Dementia Scale [25], and the Self-Maintaining and Instrumental Activities of Daily Living Scale [27].

### 2.4. Quality of Life (QoL)

QoL was assessed using the SF-36 questionnaire [28–30]. The SF-36 has four scales for physical health: physical functioning (PF, 10 items), role physical (RP, 4 items), bodily pain (BP, 2 items), and general health (GH, 5 items). It also has four scales for mental health: vitality (VT, 4 items), social functioning (SF, 2 items), role emotional (RE, 3 items), and mental health (MH, 5 items). Each subscale provides a score from 0 to 100, with higher scores indicating better perceived health. Prior work recommended analyzing the eight individual scores rather than a composite score, as only 1.8% of papers reported in a meta-analysis used a single summary measurement [31]. The SF-36 has been validated in cognitively impaired populations [32], although scores from individuals with a Mini-Mental State Examination (MMSE) score < 16 may be unreliable due to impaired insight [33].

### 2.5. Brain Regions Volume (BRV) Assessment

Brain MRI scans were obtained using a 1.5 Tesla GE Healthcare Optima MR360 scanner (General Electric Healthcare, Chicago, IL, USA). The imaging protocol included T1-weighted (TR = 7904 ms, TE = 2460 ms, field of view = 256 × 256 mm with 1 mm contiguous slices) and T2-weighted fluid attenuated inversion recovery (FLAIR; TR = 8000 ms, TE = 123 ms, inversion time = 2000 ms, field of view = 256 × 162 mm with 2 mm contiguous slices). The volumetric analysis included total brain volume, cortical thickness, white matter hyperintensities (WMH), and cerebral infarction. Exclusion criteria for MRI scans were the presence of a pacemaker, aneurysm clip, neurostimulator, cochlear implant, body weight over 110 kg, or history of primary medical conditions or procedures thought to confound WMH measurement, such as multiple sclerosis, brain radiotherapy, brain surgery, implants for Parkinson’s disease, lupus, brain tumor (lymphoma), HIV, neurocysticercosis, neurosyphilis, brain tuberculosis, and brain trauma with loss of consciousness. All clinical and neuroimaging data were collected within a three-week time interval.

Whole-brain volumes were quantified in cm^3^, defined as the number of labeled voxels multiplied by voxel dimensions. The thickness and volumes were co-registered to the brain-extracted T1-weighted volumes defined by FreeSurfer (v6.0, Surfer.nmr.mgh.harvard.edu, accessed on 18 July 2025). This software provides an array of tools for reconstructing gray/white and pial surfaces [34].

### 2.6. Statistical Analysis

We used descriptive statistics to analyze the baseline characteristics. The SF-36 domains were scored using Principal Component Analysis (PCA) with varimax rotation. We analyzed 52 variables reflecting brain volume and cortex thickness. To preserve correlation structures, these highly interrelated variables were reduced using PCA with oblique rotation (delta = 0) [35]. The computed scores were utilized for canonical regression analysis adjusted with demographic variables and presence of pre-existing health conditions [i.e., T2DM and hypertension (high blood pressure—HBP)] to explain the SF-36 score.

The PCA suitability for structure detection was tested with the Kaiser–Meyer–Olkin statistics [36] and Bartlett’s test of sphericity [37]. Because the frontal lobe is involved in creative thinking, memory, and emotional behavior, a specific analysis was performed for frontal areas separately from other brain areas.

Canonical correlation analysis identifies the linear combination of two multivariate sets (u: SF-36 domains and BRV, including sex, age, T2DM, HBP, and memory tests) that maximizes their correlation. The strength of each pair of canonical variates was calculated as the canonical root, and the procedure is repeated for the remaining unexplained variance (residuals) to generate a second canonical root, with a maximum number of canonical roots equal to the smallest canonical variate. Loadings were used to evaluate shared variance between variables and canonical composites [38]. Our analysis included the SF-36 domains (list u) correlated with the factor scores of BRV and clinical variables (list v). The significance of canonical roots was assessed using a chi-square approximation:

$$x^{2}=[-(N-(0.5\cdot (p+q+1)))]\cdot \sum_{i=1}^{s}ln\left(1-r_{ci}^{2}\right)$$

where N is the total sample size, p and q correspond to the number of variables in the two data sets, and r_ciˆ2 is the ith canonical correlation. The chi-square distribution has p × q degrees of freedom. Two sensitivity models were performed: one with individuals who had complete data sets (*n* = 299) and a second model excluding 21 patients with a diagnosis of dementia.

Each canonical root (Rci) represents a distinct dimension of variance shared by the two variable sets (u, v); meanwhile, the canonical loadings indicate the strength and direction of the association between the original variables and the canonical variates (variable contributions). The positive or negative values show the contribution in the same or opposite direction within the canonical root. The exploratory univariate analyses were performed using SPSS v.25 [39], and the inferential statistics with Stata MP 19 (StataCorp College Station, TX, USA) [40].

## 3. Results

### 3.1. Socio-Demographic and Clinical Characteristics of the Study Sample

The demographic and clinical characteristics of 420 individuals are shown in Table 1. Systolic and diastolic blood pressure (BP) were significantly higher in men than in women, but women had a higher frequency of antihypertensive treatment.

### 3.2. Quality of Life

The SF-36 scores indicated that men scored higher than women; meanwhile, memory scores showed no significant difference between sexes (Table 2). The domains were not orthogonal to each other, nor could they be differentiated into two main groups, physical and mental (Supplementary Table S1 and Supplementary Figure S1). However, we decided to maintain the scores for each dimension analyzed in canonical regression; this strategy accounts for the maximum correlation within dimensions. The PCA structure of the eight domains of the SF-36 was calculated using data from 418 subjects. The KMO value was 0.776 (Bartlett Sphericity, *p* < 0.001), suggesting the use of PCA is suitable for these data, and the two components explained 52% of the total variance. The correlation coefficients within SF-36 domains are shown in Supplementary Table S3.

### 3.3. Brain Regions (BRV)

Complete brain volume and cortex thickness data (52 measurements) were available for 315 participants. Factor analysis with oblimin rotation indicated that eight factors explained 73% of the total variance (Supplementary Table S2). The structure matrix suggests that we should maintain separate data for whole-brain and frontal lobe measurements. We calculated PCA scores from five factors representing the whole brain (total volumes of cortex, subcortical gray matter, white volume; para hippocampal and entorhinal thickness; cerebellum; insula thickness; and entorhinal volume) and four factors representing the frontal lobe (volume of orbital, middle, and superior frontal areas; thickness of the same areas; medial and lateral orbitofrontal thickness; and frontal pole). These scores explained 80.8% of the total brain variance and 67% of the frontal lobe variance (KMO = 0.84; Bartlett Sphericity, *p* < 0.001). Once these factors were obtained, the standardized residuals from intracranial volume were used for adjustment.

The correlations with oblimin rotation showed that the global brain was directly correlated with the cerebellum and hippocampus but inversely with the insula (Supplementary Table S4). Despite a significant correlation between the global brain and frontal volume, there was a lack of correlation with frontal thickness, lateral orbitofrontal thickness, and the frontal pole. The frontal lobe showed inverse correlations with insula volume and thickness. Adjustment for the intracranial volume did not affect these results. The correlation matrixes of clinical variables and brain anatomical characteristics are described in Supplementary Table S5.

### 3.4. Relationships Among Clinical Variables, Brain Regions, and SF-36 Scores

**Figure 1.**
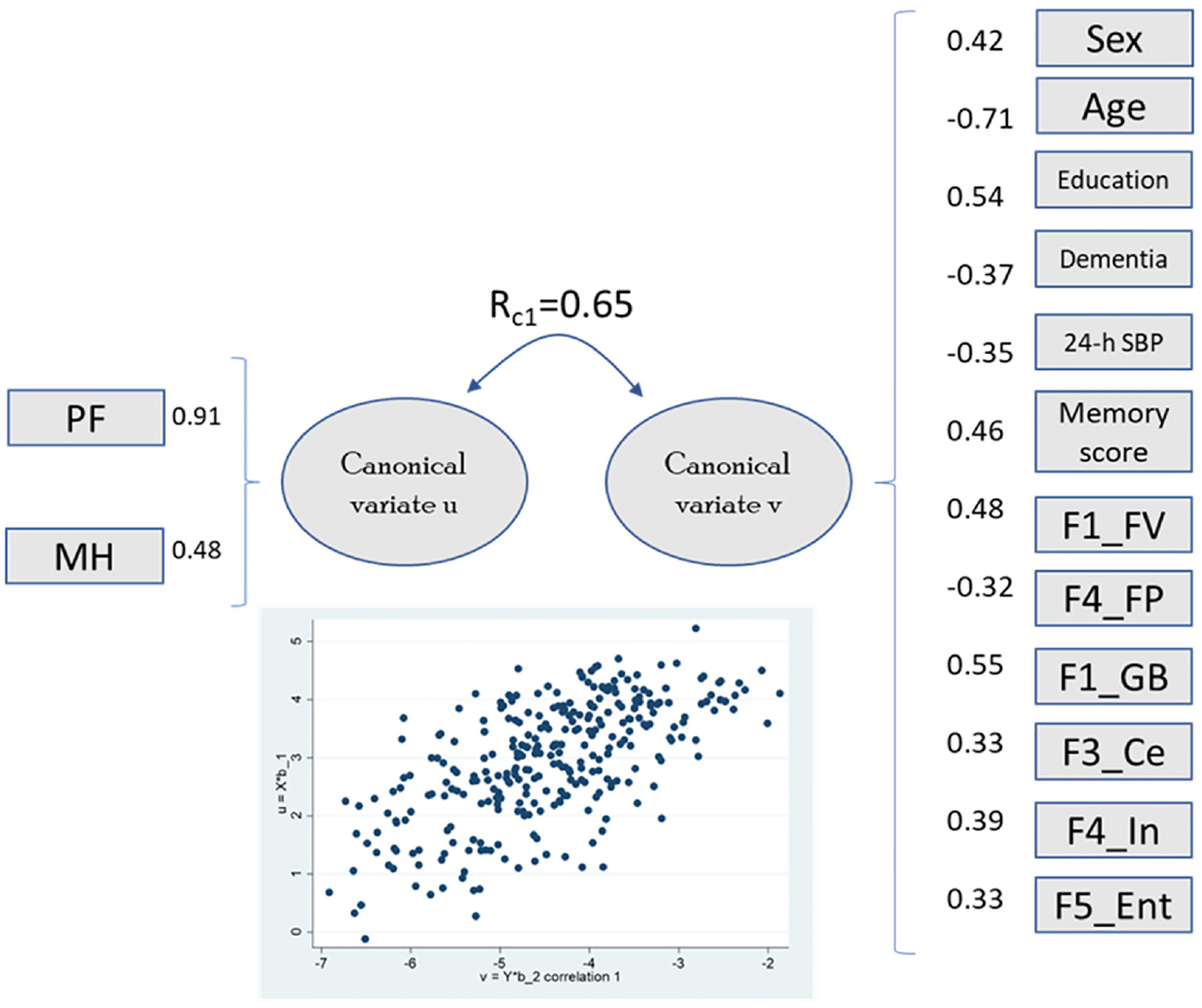
Canonical loadings greater than |0.3| and first canonical root coefficient between variates. The scatter plot shows the correlation for variates u (*y*-axis) and v (*x*-axis). Abbreviations: PF, physical function; MH, mental health; SBP, systolic blood pressure; F1_FV, frontal volume; F4_FP, frontal pole; F1_GB, global brain; F3_Ce, cerebellum; F4_In, insula; F5_Ent, entorhinal. (Complete data in *n* = 299).

**Figure 2.**
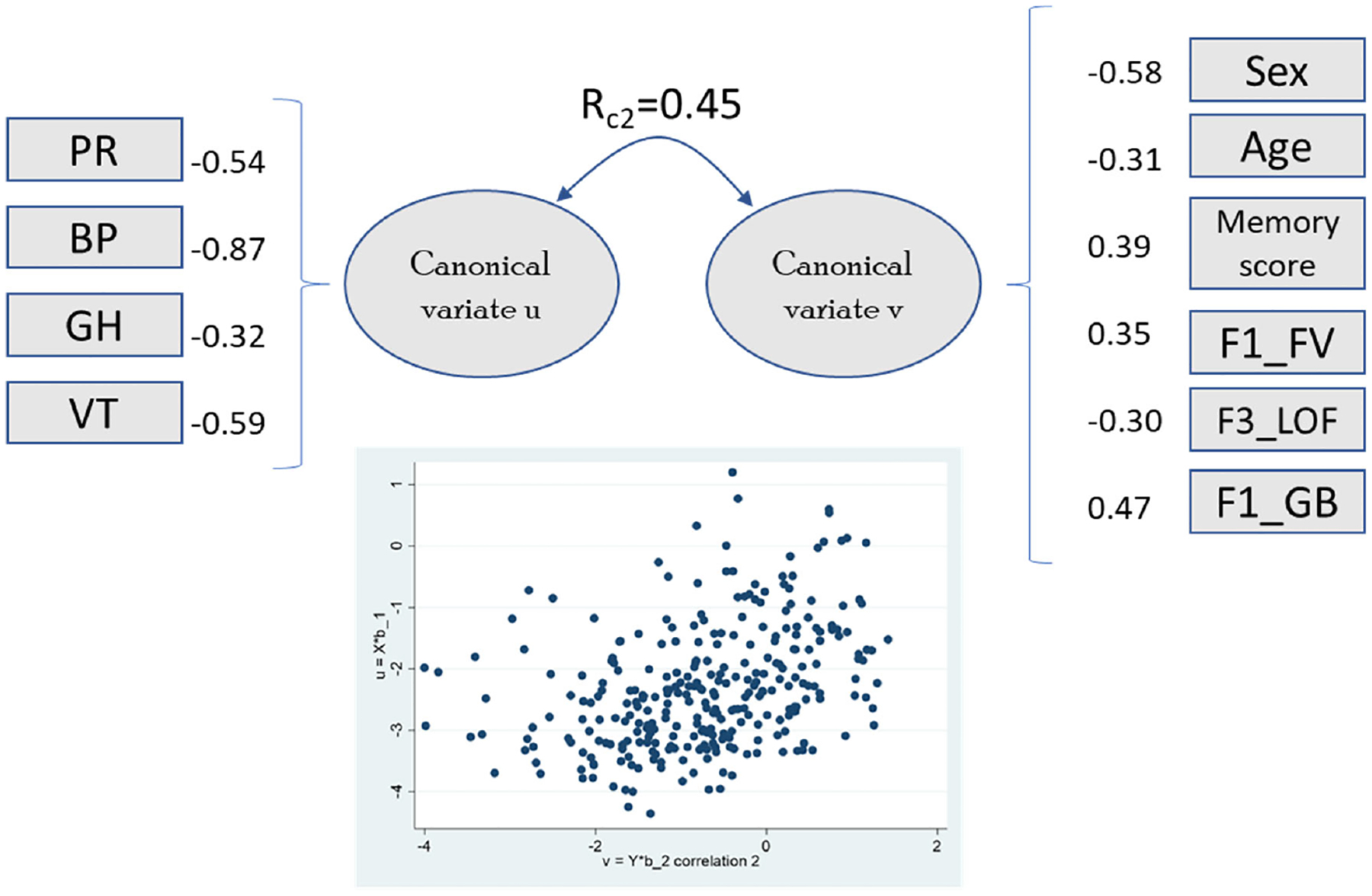
Canonical loadings greater than |0.3| and second canonical root coefficient between variates. The scatter plot shows the correlation for variates u (*y*-axis) and v (*x*-axis). Abbreviations: PR, physical role; BP, bodily pain; GH, general health; VT, vitality; F1_FV, frontal volume; F3_LOF, lateral orbitofrontal thickness; F1_GB, global brain (complete data in *n* = 299).

Loadings were computed between all independent and dependent variables (Supplementary Table S6). The first two canonical correlation roots (Rc1 = 0.65 and Rc2 = 0.45) were both statistically significant (Wilk’s lambda, *p* < 0.03; Supplementary Table S7a,b; Figures 1 and 2). The canonical correlations from Rc3 to Rc8 were 0.31, 0.26, 0.25, 0.19, 0.16, and 0.07, respectively.

The first canonical root correlates with physical function and marginally (r~0.3) with vitality and mental health, combining the two main SF-36 components. This variate was significantly related to sex, age, dementia, systolic blood pressure, and memory score. Some QoL domains were related to specific brain regions (frontal lobe, global brain, cerebellum, insula, and entorhinal areas). The second canonical root, which included physical function, general health, and vitality, was associated with global brain, frontal lobe volume, and lateral orbital frontal area. Role emotional and social functioning were not significantly associated with any brain region (the correlation between SF-36 domains and clinical/brain factors is shown in Supplementary Table S6).

### 3.5. Sensitivity Analysis and Stability of the Estimates

To test sensitivity, we computed a second model without the 21 participants with a diagnosis of dementia to analyze the modifications on the obtained loadings (Table S7a–d). When comparing these models (with and without the 21 individuals with clinical dementia), there was stability on the estimators for loading coefficients. The canonical loadings and correlation coefficients between SF-36 domains and the first two canonical variates (see Figures 1 and 2 and Supplementary Table S7a,b) are comparable to the cohort excluding those with dementia (Supplementary Table S7c,d); the first canonical root demonstrates that both physical function and mental health are statistically significant in both models. From this, it is evident that they can be used reliably for analysis with the clinical variables and anatomical brain regions in this study (those described in Supplementary Table S6).

When observing the canonical loadings between both clinical variables and brain regions and the first two canonical variates (see Supplementary Table S7a,b), there were only a few differences in figures that are of note. The first and second canonical roots demonstrate that sex, age, and education status remained stable, where they were all statistically significant in both models. When dementia patients are removed, the first canonical root in model 2 demonstrates that hypertension is no longer statistically significant (in which the canonical loading rises to *−*0.287), suggesting a link between the presence of dementia and hypertension.

## 4. Discussion

This study explored the association between QoL perception and brain region volumes and cortical thickness in elderly Hispanic adults, adjusting for sociodemographic variables and health conditions such as sex, education, and dementia. While SF-36 scores are often grouped into physical and mental health components, our findings indicate that these components may not map neatly onto distinct BRV. Notably, both physical functioning and mental health domains shared a similar direction in the first canonical root, suggesting overlapping neurobiological correlates.

### 4.1. SF-36 Mathematical Space and Variables

The SF-36 domains can be conceptualized as dimensions within a multiple structure (called a mathematical space), where each domain is represented as a vector, and the distances between them vary according to age, health status, and population characteristics. The SF-36 is reliable and stable in the Mexican adult population residing near the U.S.– Mexico border [41]. In our sample, we observed a separation of the physical functioning domain from other QoL dimensions starting at age 65. To illustrate this concept of domain proximity, we refer to prior work by Hobart et al., who visualized distances between SF-36 dimensions in patients with multiple sclerosis using multidimensional scaling [42]. Their figures (Supplementary Figure S2, upper panels) were obtained from hospital facilities (rehabilitation, outpatient clinic, and admission). This sample is different than ours, but it is clear how QoL domains can cluster or diverge under specific conditions (Supplementary Figure S2, lower panel). Although it is not a statistical comparison, the visuals reveal differences in domains across the samples.

Hispanic populations, particularly those with T2DM, have been shown to report higher QoL scores than other ethnic groups, despite higher disease burdens [43]. However, aging and comorbidities, such as T2DM, tend to lower QoL scores, especially in physical domains, indicating the need to consider disease-specific and cultural perception modifiers. Additionally, socioeconomic factors impact cognitive development and school performance. Studies in pediatric populations have shown that latent factors like income, educational environment, and physiological stress correlate with cortical surface area and cognitive outcomes [44], which may persist and modulate QoL perception later in life.

### 4.2. Brain Regions Approach

The separate factor analysis of frontal brain regions we performed was justified by their prominent role in emotion and executive function. The prefrontal cortex, particularly in humans, is expanded and deeply involved in social behavior and goal-directed cognition [45]. Emotional networks include the orbitofrontal cortex, medial prefrontal cortex (MPFC), inferior frontal gyri, superior temporal sulci, temporal poles, and cerebellum—all regions associated with empathic and affective processing [46].

We used oblimin rotation to reflect the possible functional interdependence of BRV, which enhances computational flexibility and reduces collinearity. The calculated factor scores represent statistical composites that require careful interpretation. The biological specificity of these components must be validated in external datasets. The known biological functions are supported by clinical observations in patients with brain tumors or metastases, where overlapping physical, cognitive, and emotional symptoms emerge. Our study focused on structural MRI to capture anatomical variations across regions. The use of structural markers, such as regional brain volume and cortical thickness, offers a complementary perspective on the neurobiological substrates of perceived quality of life.

### 4.3. QoL as a Neuro-Social Marker and Pharmacoeconomic Surrogate and Sensitivity Analysis

Canonical correlation analysis revealed complex associations between QoL domains and clusters of neuroanatomical, demographic, and clinical variables. Notably, the first canonical root (Rc1 = 0.65) linked physical functioning and mental health domains with male sex, higher education, and volumes in frontal, global brain, cerebellar, and entorhinal regions. These regions support executive function, emotional regulation, coordination, and memory. In contrast, older age, dementia, and higher factor scores for parietal and insular regions contributed negatively, suggesting these factors diminish QoL by reducing cognitive and affective integration.

The second canonical root (Rc2 = 0.45) reflected associations between remaining physical domains (physical role, bodily pain, general health) and vitality, correlating with older age, male sex, and orbitofrontal volume. These findings are consistent with the known role of the ventral attention system (integrates input from the frontal cortex, basal ganglia, and temporoparietal junction) in attentional control and behavioral flexibility [47,48]. Recent evidence has demonstrated that the cerebellum is related to motor and non-motor functions, including emotion and cognition. Alterations in these areas have been recognized in early stages of Alzheimer’s disease [49].

Our findings support the view of QoL as a neuro-social marker that reflects the integrated influence of brain structure, cognitive function, and social factors such as education and sex. QoL is not a subjective matter isolated from biology; it emerges as a bridge of neurological integrity and social context.

Although this study did not include an economic evaluation, our findings suggest that QoL could function as a clinical, patient-centered outcome with utility in pharmacoeconomics. With the use of quality-adjusted life years (QALYs), QoL may inform broader pharmacoeconomic frameworks by capturing how individuals perceive the impact of disease and interventions on their daily functioning and well-being.

Interestingly, when participants with dementia were excluded in the sensitivity analysis, the loadings for most brain regions on Rc1 increased, while for Rc2, there were variable responses. Therefore, individuals with cognitive impairment contribute to the overall variance captured in these roots, amplifying the brain–QoL relationship through more pronounced structural changes and lower perceived well-being. Their inclusion in the main analysis reflects a broader neurocognitive spectrum and enhances the ecological validity (real-life applicability) of findings relevant to aging Hispanic populations [50].

### 4.4. Cultural Considerations

The Hispanic community living in the US, particularly in the U.S.–Mexico border, experiences an intersection of cultural identity and health disparities that can influence QoL perceptions [51]. Conditions like diabetes and heart failure tend to have worse clinical outcomes in Hispanic individuals, yet paradoxically, they often report higher QoL than their non-Hispanic counterparts [52]. This may stem from cultural resilience, social networks, or other unmeasured psychological traits [53]. Although cultural values are important, we did not include direct measures of social support or belief. These unmeasured variables can be mediators of the relationship between disease burden, brain structure, and QoL.

Our findings on canonical roots may reflect cultural strengths, such as familism, community interdependence, spiritual values, and coping strategies that contribute to emotional resilience. The SF-36 requires respondents to make abstract self-evaluations of their behaviors and life goals. These processes engage the medial prefrontal cortex (mPFC), particularly areas involved in intentionality and metacognition [54]. The integration of behavioral, cognitive, and affective processing in these regions supports our findings of shared influences across QoL domains.

### 4.5. Strengths and Limitations

This study has several limitations. Its cross-sectional design precludes causal inference. The use of structural MRI limits the ability to infer functional connectivity or temporal dynamics. Additionally, our sample was limited to Hispanic adults from a single geographic region, reducing generalizability. The canonical models explained 42% and 20% of variance across domains, but individual loadings for brain region volume and cortical thickness were modest (11–23%). Further studies are needed to explore the link between hypertension and vascular dementia, which may also impact QoL and cognitive decline.

Nevertheless, our sample size provided adequate statistical power, and the inclusion of sensitivity analyses—excluding dementia cases and adjusting for intracranial volume—reinforced the robustness of findings. Notably, we observed consistent associations between brain regions (e.g., cerebellum, insula, entorhinal cortex) and cognitive or QoL domains, which align with current literature.

## 5. Conclusions

We conclude that perceived QoL is not merely a subjective experience evaluation but a complex combination of biological and social variables. Age, hypertension, and brain structural changes shape perceptions of well-being, independence, and cognitive function. We provide evidence that these factors jointly influence QoL in Hispanic older adults. This multidimensional understanding of subjective health perception may contribute to the development of economic outcomes, where QoL assessment remains a central component in health policy and pharmacoeconomic frameworks for cost-effectiveness perspectives. Expanding this research to include diverse racial, cultural, and socioeconomic populations will deepen our understanding of global life satisfaction and its neurobiological foundations.

## Supplementary Material

Supplementary

**Supplementary Materials:** The following supporting information can be downloaded at https://www.mdpi.com/article/10.3390/ctn9030033/s1: Supplementary Table S1: Structure of principal components with two factors, using oblimin rotation. Supplementary Table S2. Principal component analyses for the brain region. Supplementary Table S3. Correlation coefficients among SF-36 domains. Supplementary Table S4. Correlations among brain regions. Supplementary Table S5. Correlations among clinical variables and anatomical brain factors. Supplementary Table S6. Correlation between SF-36 domains and clinical/brain factors. Supplementary Table S7a. Canonical loadings and correlation coefficients between SF-36 domains. Supplementary Table S7b. Canonical loadings and correlation coefficients between clinical variables and anatomical brain factors adjusted by intracranial volume. Supplementary Table S7c. Canonical loadings and correlation coefficients between SF-36 domains and the first two canonical variates in individuals without dementia. Supplementary Table S7d. Canonical loadings and correlation coefficients between clinical variables and anatomical brain factors adjusted by intracranial volume in individuals without dementia. Supplementary Figure S1. Plot of distances of principal components of the SF-36 domains. Supplementary Figure S2. Plot of distances of principal components comparison between Hobart et al. (Ref. [42]) and our study.

## Figures and Tables

**Table 1.** Baseline characteristics of the total sample, women, and men.

Baseline Characteristics (Units)	Total	Women	Men	*p* *
	(*n* = 420)	(*n* = 300)	(*n* = 120)	
Age (y)	56.1 ± 11.5	55.8 ± 11.2	57.1 ± 12.4	0.287
Education (y)	6.8 ± 4.5	6.5 ± 4.5	7.3 ± 4.6	0.093
BMI, kg/m^2^	28.8 ± 5.8	29 ± 5.9	28.3 ± 5.6	0.226
Waist circumference (cm)	91.4 ± 12.1	89.8 ± 11.9	95.5 ± 11.9	<0.001
Systolic BP (mmHg)	139.5 ± 24.1	137.2 ± 24.2	145.3 ± 23	0.002
Diastolic BP (mmHg)	79.3 ± 10.8	77.3 ± 10.2	84.4 ± 10.5	<0.001
Obesity (% of sample)	39 [164/416]	41.3 [123]	34.7 [41]	0.219
Diabetes mellitus (%)	12 [52/418]	11.7 [35]	14.3 [17]	0.471
24-h hypertension (%)	52 [218/418]	50.2 [150]	57.1 [68]	0.198
Use of antihypertensive treatment (%)	29 [123/418]	34 [101]	19 [22]	0.002
History of stroke (%)	2 [8/416]	2 [6]	1.7 [2]	0.82
Dementia at baseline (%)	7 [29/416]	6.4 [19]	8.4 [10]	0.468
Type of dementia at baseline (%)				
Alzheimer’s disease (AD)	1.4 [6/416]	1.7 [5]	0.8 [1]	0.515
Vascular dementia (VD)	4.3 (18/416)	3.4 (10)	6.7 (8)	0.128
AD + VD	0.7 (3/416)	1.7 (2)	0.3 (1)	0.143

Abbreviations: BMI, body mass index; BP, blood pressure. Continuous variables are mean ± SD; nominal variables are % of total sample [*n* = number of affected individuals]. The total column denotes the percentage [n/N].

* *p* values were obtained by Student’s *t*-test or *χ*^2^.

**Table 2.** Memory scores and quality of life assessed by SF-36 for the total sample, women, and men.

Variables	Total	Women	Men	*p* *
	(*n* = 420)	(*n* = 300)	(*n* = 120)	
Memory score	18.5 ± 4.9	18.3 ± 4.8	18.9 ± 5.2	0.249
Orientation score	9.1 ± 1.4	9.1 ± 1.4	8.98 ± 1.4	0.30
SF-36 domain				
Physical functioning	62.5 ± 24.4	57.4 ± 23.4	75.3 ± 22.3	<0.001
Physical role	80.1 ± 37.9	77.4 ± 39.6	87 ± 32.5	0.012
Bodily pain	65 ± 22.3	63.6 ± 21.3	68.6 ± 24.5	0.042
General Health	72.9 ± 17.6	72.5 ± 17.2	74.1 ± 18.6	0.408
Vitality	53.1 ± 12.7	51.6 ± 11.8	57 ± 14	<0.001
Social functioning	92 ± 17.7	91.9 ± 17.4	92.1 ± 18.5	0.921
Role emotional	80.6 ± 36.6	77 ± 38.8	89.6 ± 28.7	0.001
Mental health	62.6 ± 14.3	61.1 ± 14.4	66.5 ± 13.3	<0.001

Data are mean ± SD.

* *p* values were calculated using Student’s *t*-test adjusted by variance.

## Data Availability

The data utilized in this study are derived from the Maracaibo Aging Study. The data collected, as well as the statistical coding of the analysis, will be available from the corresponding author upon reasonable request.

## Abbreviations

AD: Alzheimer’s Disease
AD + VD: Alzheimer’s Disease + Vascular Dementia
APC: Article Processing Charge
BMI: Body Mass Index
BP: Blood Pressure
BRV: Brain Regions Volume
FLAIR: Fluid Attenuated Inversion Recovery
GH: General Health
HBP: High Blood Pressure
KMO: Kaiser–Meyer–Olkin
MAS: Maracaibo Aging Study
MH: Mental Health
MMSE: Mini-Mental State Examination
MRI: Magnetic Resonance Imaging
mPFC: Medial Prefrontal Cortex
PCA: Principal Component Analysis
PF: Physical Functioning
QoL: Quality of Life
QALYs: Quality-Adjusted Life Years
RE: Role Emotional
RP: Role Physical
SBP: Systolic Blood Pressure
SD: Standard Deviation
SF: Social Functioning
SF-36: Rand’s 36-Item Short Form Survey
T2DM: Type 2 Diabetes Mellitus
VT: Vitality
VD: Vascular Dementia
WMH: White Matter Hyperintensities
