# Supplementary material for: Perception of Quality of Life, Brain Regions, and Cognitive Performance in Hispanic Adults: A Canonical Correlation Approach
Source: Clin Transl Neurosci. Author manuscript; Available in PMC 2026 Apr 7. (PMC13052506; doi:10.3390/ctn9030033)
Supplement: Supplementary [file NIHMS2161954-supplement-Supplementary.pdf]

Supplementary Table 1. Structure of principal components with two factors, using oblimin rotation. The KMO was 0.78, and the two components explained 52% of total variance.

**SF-36 domain**	**Mental component**	**Physical component**
Physical Functioning	0.12	**0.84**
Role Physical	0.46	**0.51**
Bodily Pain	**0.63**	0.44
General Health	**0.54**	**0.55**
Vitality	**0.72**	0.38
Social Functioning	**0.76**	-0.04
Role Emotional	**0.58**	0.21
Mental Health	**0.78**	0.13

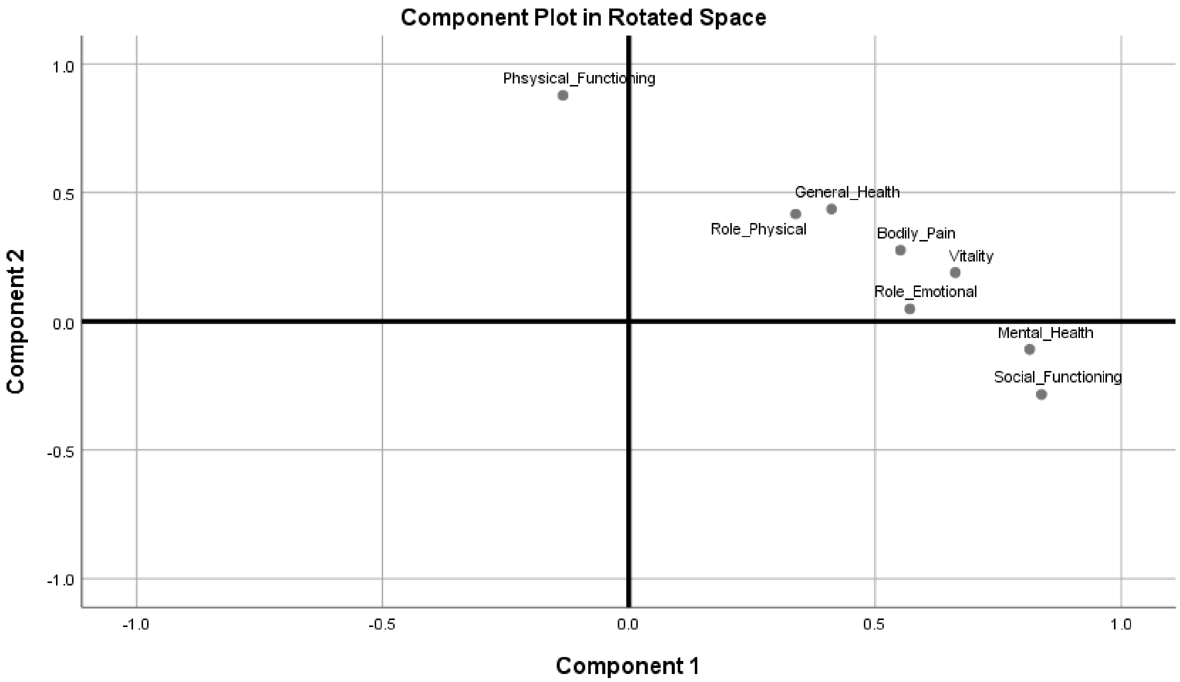

Supplementary Figure 1. Plot of distances of principal components of the SF-36 domains. The axes were oblimin rotated with a delta parameter = 0.

Supplementary Table 2. Principal component analyses for brain parcellation.

Brain parcellation	Components							
	1	2	3	4	5	6	7	8
Cortex volume	0.970							
Right cortex volume	0.967							
Left cortex volume	0.966							
Supra-tentorial volume	0.948			0.543				
Total gray volume	0.942			0.587				
Brain segment volume	0.929			0.633				
Left superior frontal volume	0.868							
Right superior frontal volume	0.868							
Left cortical white matter volume	0.854			0.576				
Sub-cortical gray volume	0.854							
Cortical white matter volume	0.851			0.583				
Left lateral-orbito-frontal volume	0.844							
Right cortical white matter volume	0.843			0.586				
Left rostral-middle-frontal volume	0.820							
Left insula volume	0.819							
Right lateral-orbito-frontal volume	0.814							
Right insula volume	0.812							
Right rostral-middle-frontal volume	0.762							
Left caudal-middle-frontal volume	0.724							
Right medial-orbito-frontal volume	0.697							
Right caudal-middle-frontal volume	0.640							
Left hippocampus	0.593							
Right hippocampus	0.573							-0.535
Left medial-orbito-frontal volume	0.537							
Left caudal-middle-frontal thickness		0.763						
Right caudal-middle-frontal thickness		0.785						
Right superior frontal thickness		0.854						
Left superior frontal thickness		0.865						
Right rostral-middle-frontal volume		0.801						
Left rostral-middle-frontal volume		0.766						
Left lateral-orbito-frontal thickness			0.767					
Right lateral-orbito-frontal thickness			0.728					
Left medial-orbito-frontal thickness			0.775					
Right medial-orbito-frontal thickness			0.761					
Left cerebellum white matter				0.854				
Right cerebellum cortex				0.896				
Left cerebellum cortex				0.889				
Right cerebellum white matter				0.863				
Left parahippocampal thickness					0.825			
Right parahippocampal thickness					0.812			
Right parahippocampal volume					0.686			
Left parahippocampal volume					0.757			
Right frontalpole volume						-0.732		
Left frontalpole volume						-0.762		
Right frontalpole thickness						-0.752		
left frontalpole thickness						-0.708		
Right entorhinal volume							-0.574	
Left entorhinal volume							-0.621	
Left entorhinal thickness							-0.805	
Right entorhinal thickness							-0.751	
Left insula thickness								
Right insula thickness								

Rotated structured matrix using oblimin technique with delta=0 to obtain adequate flexibility for correlated factors.
Values are only shown for brain anatomical areas explaining more than 50% of the variance.
A total of eight factors are explained by the brain anatomical areas included in the factor analyses.

Supplementary Table 3. Correlation coefficients among SF-36 domains

SF-36 domains	**Physical functioning**	**Physical role**	**Bodily pain**	**General health**	**Vitality**	**Social functioning**	**Role emotional**	**Mental health**
Physical functioning	–							
Physical role	0.258	–						
Bodily pain	0.299	0.482	–					
General health	0.298	0.267	0.348	–				
Vitality	0.401	0.396	0.356	0.444	–			
Social functioning	0.082	0.357	0.237	0.179	0.305	–		
Role emotional	0.235	0.352	0.235	0.227	0.349	0.544	–	
Mental health	0.295	0.291	0.297	0.331	0.531	0.386	0.451	–

Values greater than 0.1 are $p<0.05$

Supplementary Table 4. Correlations among brain regions

	GB	PH	Ce	In	Ent	FV	FT	LOF	FP
GB	–								
PH	0.285	–							
Ce	0.437	0.187	–						
In	-0.261	-0.230	-0.067	–					
Ent	0.264	0.269	0.100	-0.205	–				
FV	0.916	0.282	0.368	-0.385	0.290	–			
FT	-0.110	0.245	0.010	-0.476	0.014	0.003	–		
LOF	-0.018	0.044	0.071	-0.311	0.135	0.075	0.116	–	
FP	-0.082	-0.198	-0.039	0.207	-0.055	-0.067	-0.215	-0.366	–

Abbreviations: GB, global brain; PH, parahippocampus area; Ce, cerebellum; In, insula; Ent, entorhinal; FV, frontal volume; FT, frontal thickness; LOF, lateral orbitofrontal thickness; FP: frontal pole.

Correlations were adjusted by intracranial volume.

Values greater than 0.1 are $p<0.05$

Supplementary Table 5. Correlations among clinical variables and anatomical brain factors.

	Education	Sex	Age	BMI	Dementia	systolic BP	Memory score	DM	FV	FT	LOF	FP	GB	PH	Ce	In	Ent
Education	–																
Sex	0.10	–															
Age	-0.51	0.10	–														
BMI	0.16	-0.06	-0.17	–													
Dementia	-0.32	0.04	0.35	-0.12	–												
Systolic BP	-0.12	0.18	0.34	0.07	0.112	–											
Memory score	0.64	0.03	-0.38	0.16	-0.470	-0.10	–										
DM	-0.04	0.06	0.14	0.10	-0.026	0.20	-0.02	–									
FV	0.28	0.19	-0.43	0.02	-0.309	-0.11	0.31	0.02	–								
FT	0.14	-0.23	-0.40	-0.02	-0.216	-0.21	0.16	-0.11	0.32	–							
LOF	0.08	0.18	-0.03	-0.10	-0.050	0.04	0.10	0.02	0.17	0.12	–						
FP	-0.04	0.10	0.15	0.06	0.064	0.10	-0.03	-0.03	-0.06	-0.23	-0.38	–					
GB	0.23	0.18	-0.46	0.06	-0.301	-0.10	0.29	-0.01	0.81	0.18	0.05	-0.10	–				
PH	0.16	-0.10	-0.29	0.03	-0.174	-0.21	0.11	-0.08	0.26	0.29	0.06	-0.21	0.26	–			
Ce	0.16	0.01	-0.23	0.03	-0.114	-0.06	0.15	-0.17	0.11	0.14	0.11	-0.04	0.19	0.13	–		
In	-0.27	0.003	0.42	0.08	0.310	0.17	-0.27	0.05	-0.51	-0.51	-0.32	0.20	-0.36	-0.22	-0.05	–	
Ent	0.16	0.28	-0.13	-0.13	-0.084	-0.13	0.08	-0.08	0.26	0.05	0.15	-0.06	0.21	0.23	0.04	-0.20	–

Abbreviations: BMI, body mass index; BP, blood pressure; DM, diabetes mellitus; GB, global brain; PH, parahippocampus area; Ce, cerebellum; In, insula; Ent, entorhinal; FV, frontal volume; FT, frontal thickness; LOF, lateral orbitofrontal thickness; FP: frontal pole. Values greater than 0.1 are $p<0.05$

Supplementary Table 6. Correlation between SF-36 domains and clinical/brain factors

Variables	Physical functioning	Physical role	Bodily pain	General health	Vitality	Social functioning	Role emotional	Mental health
Sex	0.303	0.167	0.236	0.091	0.269	0.115	0.140	0.245
Age	-0.388	0.027	0.072	-0.068	-0.035	0.004	0.021	-0.173
BMI	-0.088	0.005	-0.029	0.031	0.012	-0.046	-0.042	0.041
Education	0.278	0.016	-0.013	0.091	0.112	0.070	0.086	0.256
Dementia	-0.211	-0.063	-0.001	-0.057	-0.101	-0.061	-0.022	-0.140
Systolic BP	-0.210	0.019	0.015	-0.005	0.036	0.081	0.047	-0.018
Memory score	0.214	-0.026	-0.100	0.039	0.039	0.062	0.076	0.202
DM	-0.205	-0.008	-0.054	-0.127	-0.009	0.015	-0.073	-0.038
FV	0.241	-0.047	-0.085	0.052	0.069	0.073	0.109	0.200
FT	0.100	-0.016	-0.032	0.017	-0.086	0.055	0.045	-0.050
LOF	0.161	0.047	0.130	0.114	0.086	-0.016	-0.018	0.091
FP	-0.216	0.045	-0.018	-0.059	-0.055	0.066	-0.026	-0.024
GB	0.271	-0.081	-0.134	-0.009	0.041	0.051	0.075	0.179
PH	0.092	-0.045	-0.004	0.002	-0.008	-0.005	-0.008	0.077
Ce	0.139	-0.062	-0.037	-0.020	-0.077	-0.060	-0.050	0.105
In	-0.251	-0.025	0.023	-0.129	-0.071	-0.122	-0.115	-0.098
Ent	0.206	0.008	0.120	0.085	0.125	-0.043	0.065	0.168

Abbreviations: BMI, body mass index; BP, blood pressure; DM, diabetes mellitus; GB, global brain; PH, parahippocampus area; Ce, cerebellum; In, insula; Ent, entorhinal; FV, frontal volume; FT, frontal thickness; LOF, lateral orbitofrontal thickness; FP: frontal pole. Values greater than 0.1 are $p<0.05$

Supplementary Table 7a. Canonical loadings and correlation coefficients between SF-36 domains and the two first canonical variates (complete data in n=299)

	Loadings		Correlations	
SF-36 domains	1	2	1	2
Physical functioning	0.913*	-0.261	0.596	-0.103
Physical role	0.063	-0.535*	0.041	-0.212
Bodily pain	0.095	-0.870*	0.062	-0.344
General Health	0.201	-0.319*	0.132	-0.126
Vitality	0.291	-0.585*	0.190	-0.231
Social functioning	0.054	-0.022	0.036	-0.009
Role emotional	0.141	-0.055	0.092	-0.022
Mental health	0.48*	-0.162	0.314	-0.064

*Canonical loadings > |0.3|.

Supplementary Table 7b. Canonical loadings and correlation coefficients between clinical variables, anatomical-brain factors adjusted by intracranial volume and the two first canonical variates (complete data in n=299)

Variables	Loadings		Correlations	
	1	2	1	2
Sex	0.424*	-0.575*	0.277	-0.228
Age	-0.709*	-0.310*	-0.464	-0.123
BMI	-0.095	0.007	-0.062	0.003
Education	0.536*	0.161	0.350	0.064
Dementia	-0.365*	-0.018	-0.239	-0.003
24-h systolic BP	-0.348*	-0.092	-0.228	-0.036
Memory score	0.458*	0.390*	0.299	0.154
DM	-0.289	0.015	-0.188	0.006
F1_FV	0.480*	0.352*	0.314	0.139
F2_FT	0.140	0.234	0.091	0.093
F3_LOF	0.234	-0.303*	0.153	-0.120
F4_FP	-0.315*	0.039	-0.206	0.016
F1_GB	0.548*	0.467*	0.358	0.185
F2_PH	0.201	0.099	0.131	0.039
F3_Ce	0.327*	0.244	0.214	0.097
F4_In	-0.394*	-0.185	-0.258	-0.073
F5_Ent	0.333*	-0.257	0.218	-0.102

Abbreviations: BMI, body mass index; BP, blood pressure; DM, diabetes mellitus; F1_GB, global brain; F2_PH, parahippocampus area; F3_Ce, cerebellum; F4_In, insula; F5_Ent, entorhinal; F1_FV, frontal volume; F2_FT, frontal thickness; F3_LOF, lateral orbitofrontal thickness; F4_FP: frontal pole.
*Canonical loadings > |0.3|.

Supplementary Table 7c. Canonical loadings and correlation coefficients between SF-36 domains and the two first canonical variates in individuals without dementia (n=278)

	Loadings		Correlations	
SF-36 domains	1	2	1	2
Physical functioning	0.937*	-0.233	0.597	-0.102
Physical role	0.089	-0.623*	0.057	-0.273
Bodily pain	0.155	-0.816*	0.099	-0.357
General Health	0.205	-0.382*	0.131	-0.167
Vitality	0.344*	-0.662*	0.219	-0.290
Social functioning	0.043	-0.176	0.075	-0.077
Role emotional	0.142	-0.223	0.090	-0.098
Mental health	0.446*	-0.133	0.284	-0.058

*Canonical loadings >|0.3|.

Supplementary Table 7d. Canonical loadings and correlation coefficients between clinical variables and anatomical brain factors adjusted by intracranial volume, and the two first canonical variates in individuals without dementia (n=278)

	Loadings		Correlations	
**Variables**	**1**	**2**	**1**	**2**
Sex	0.567*	-0.551*	0.361	-0.241
Age	-0.615*	-0.417*	-0.392	-0.183
BMI	-0.166	0.001	-0.106	0.001
Education	0.463*	0.20	0.295	0.088
Dementia (Excluded n=21)	NA	NA	NA	NA
24-h systolic BP	-0.287	-0.201	-0.183	-0.088
Memory score	0.320*	0.414*	0.204	0.181
DM	-0.328*	0.022	-0.209	0.010
FV	0.346*	0.353*	0.221	0.155
FT	0.009	0.163	-0.006	0.071
LOF	0.206	-0.244*	0.131	-0.107
FP	-0.256	0.018	-0.163	0.008
GB	0.418*	0.520*	0.266	0.228
PH	0.092	0.188	0.059	0.082
Ce	0.276	0.367*	0.176	0.161
In	-0.249	-0.073	-0.159	-0.032
Ent	0.290	-0.199	0.185	-0.087

Abbreviations: BMI, body mass index; BP, blood pressure; DM, diabetes mellitus; GB, global brain; PH, parahippocampus; Ce, cerebellum; In, insula; Ent, entorhinal; FV, frontal volume; FT, frontal thickness; LOF, lateral orbitofrontal thickness; FP: frontal pole.
*Canonical loadings >|0.3|.

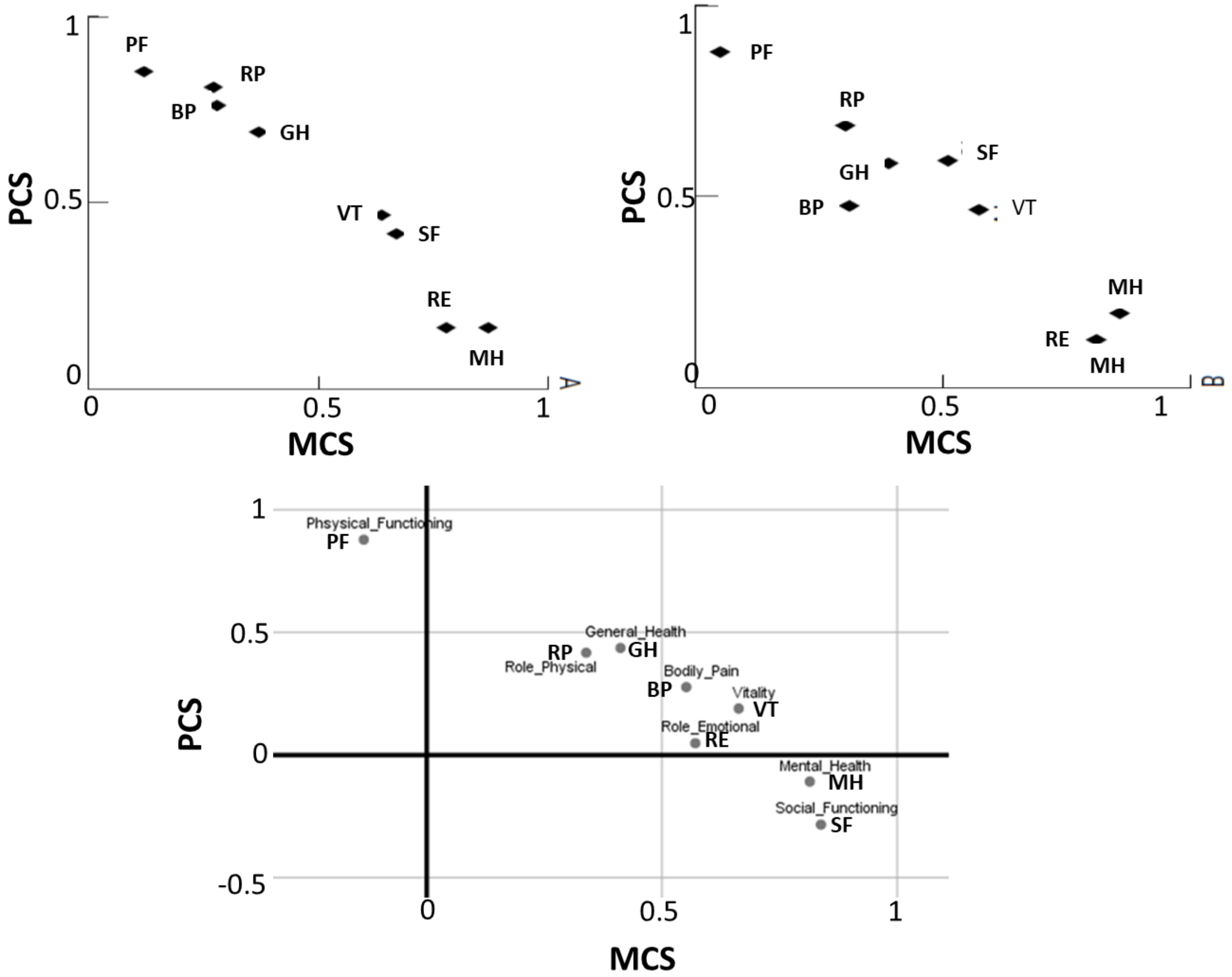

Supplementary Figure 2. The upper panels are the domains (PCS: Physical, MCS: Mental) in patients (left) and controls (right) from Hobart et al [Ref 39]. We transposed the original charts to compare the distances obtained in our study (lower center panel). The main differences are the Physical function (PF) and Social Function (SF). Physical Functioning (PF), role-physical (RP), bodily pain (BP), general health (GH), vitality (VT), social functioning (SF), role-emotional (RE), and mental health (MH).
